# Necroptosis as a key player in cervical cancer progression and management: a systematic review and transcriptomic cross-study consensus analysis

**DOI:** 10.1007/s10495-026-02440-w

**Published:** 2026-10-11

**Authors:** Seth-Frerich Fobian, Eric Eldering, Jacqueline Tromp, Arlene L. Oei

**Affiliations:** 1 Department of Radiation Oncology, Amsterdam UMC, Amsterdam, The Netherlands; 2 Laboratory for Experimental Oncology and Radiobiology (LEXOR), Cancer Center Amsterdam, Amsterdam UMC and University of Amsterdam, Amsterdam, The Netherlands; 3 Cancer Biology and Immunology, Cancer Center Amsterdam, Amsterdam, The Netherlands; 4 Department of Experimental Immunology, Cancer Center Amsterdam, Amsterdam Institute for Infection and Immunity, Lymphoma and Myeloma Center Amsterdam (LYMMCARE), Amsterdam University Medical Centers, University of Amsterdam, Amsterdam, The Netherlands; 5 Department of Medical Oncology, Amsterdam University Medical Centers, Amsterdam, The Netherlands

**Keywords:** Necroptosis, Cervical cancer, Programmed cell death, Prognostic transcriptomics, Immunogenic cell death, Interactome

## Abstract

Immunotherapy has improved outcomes for a subset of patients with locally advanced cervical cancer. However, many tumors remain immunologically “cold” and fail to respond. Strategies that enhance tumor immunogenicity are therefore of increasing interest. Necroptosis, a regulated form of inflammatory cell death, has emerged as a potential mechanism capable of promoting the anti-tumor immune responses through the release of damage-associated molecular patterns and enhanced antigen presentation. In this study, we performed a systematic review and transcriptomic cross-study consensus analysis of all studies carried out describing the role and mechanisms of necroptosis occurrence in cervical cancers. We analyzed all published necroptosis-related prognostic gene signatures and integrated these findings into an exploratory transcriptomic analyses to identify mechanistic insights into interlinked biological pathways, and potential therapeutic targets. Following PRISMA guidelines, we analyzed seven published prognostic models, from studies using RNA sequencing and least absolute shrinkage and selection operator (LASSO)-based feature selection to generate a prognostic risk score for patients. We then conducted a comparative analyses of eight overlapping genes and their reported risk score coefficients. Across studies, seven necroptosis-related genes were identified, many of which are functionally linked to mitochondrial transport and permeability regulation, reactive oxygen species (ROS) generation, and inflammatory signaling. Overall, our findings support a key role for necroptosis in cervical cancer, where activation thereof is generally associated with enhanced immune infiltration and activation; thereby improved prognosis. These findings position necroptosis as a promising target for combination therapy strategies aimed at converting immunologically “cold” tumors into more responsive phenotypes. By integrating published signatures with exploratory analyses, we identify potential avenues to modulate necroptotic signaling in ways that may enhance synergy with immunotherapies, and have synthesized a hypothetical cell signaling and interaction diagram. Collectively, this work provides a foundation for future experimental validation and therapeutic development.

**Supplementary Information:**

The online version contains supplementary material available at https://doi.org/10.1007/s10495-026-02440-w.

## Introduction

### General introduction

Despite recent advances in immunotherapy, a substantial proportion (> 80%) of cervical cancer patients remain unresponsive, often due to a poorly infiltrated or immunologically ‘cold’ tumor microenvironment [1]. Strategies that enhance tumor immunogenicity and promote immune cell recruitment are therefore of increasing interest.

**Fig. 1:**
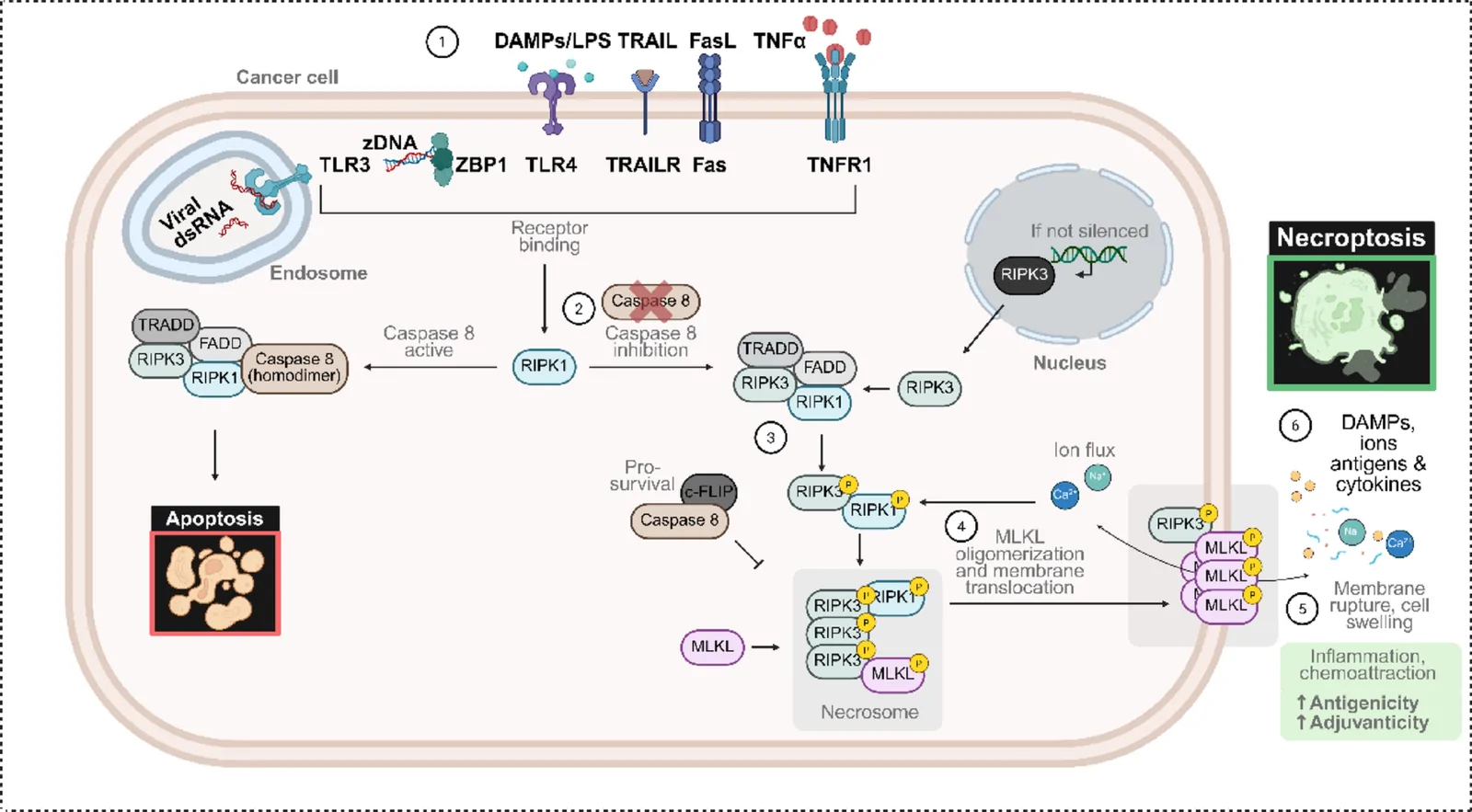
The necroptosis process overview. (1) Ligands binding to receptors and sensors trigger downstream signaling. (2) Upon caspase 8 inhibition, necroptosis is allowed to occur and apoptosis is not activated. (3) The necrosome forms and is phosphorylated (activated). (4) MLKL is phosphorylated and undergoes oligomerization and membrane translocation, creating a permeable membrane pore. (5) Cells swell and rupture. (6) DAMPs are released, leading to an increase in inflammation, antigen release, and immune cell recruitment.

Programmed cell death (PCD) pathways play a central role in shaping anti-tumor immune responses [2]. Apoptosis, the most widely studied PCD pathway, is generally considered immunologically silent [3]. In contrast, necroptosis is a regulated, necrosis-like form of PCD, characterized by membrane rupture and the release of intracellular contents, including damage-associated molecular pattern (DAMP) molecules [4]. This process promotes local inflammation, enhances antigen presentation, and stimulates immune cell recruitment [5]. At the molecular level, necroptosis is characterized by the phosphorylation (activation) of RIPK1 and RIPK3, formation of a signaling complex, and the phosphorylation and oligomerization of MLKL, forming a pore which disrupts the cell membrane, releasing DAMPs and intracellular contents (Fig. 1). Strict regulation of this pathway is essential maintaining homeostasis. For a detailed discussion of the molecular steps governing necroptosis, readers are referred to in-depth mechanistic reviews as summarized by Vandenabeele and colleagues [3, 6].

Although initially described as an alternative to apoptosis, necroptosis is now recognized as a tightly regulated and context-dependent process rather than a binary switch between cell death pathways [6]. Necroptosis is activated partly based on when apoptosis signaling is impaired, e.g. through inhibition of caspase-8. The bidirectional anti-necroptotic role of caspase-8 has been widely studied. Briefly, the formation of caspase-8 homodimers drives apoptosis, whereas the formation of caspase-8-FLIP heterodimers suppresses RIPK3-mediated necroptosis and thus promotes cell survival [7, 8].

Of all PCD pathways, necroptosis shows the strongest correlation to CD8 + T cells in the tumor microenvironment (TME), interferon (IFN) response, and cytolytic activity [9]. Also thought to have developed as a mechanism of detecting and neutralizing pathogens [10], necroptosis can have context-dependent pros and cons in the context of cancer [11]. Its occurrence is favorable: attracting immune cells to immunologically cold tumors, synergizing with immunotherapies, and fighting infection, or unfavorable: the unregulated balance of inflammation in highly inflamed TMEs potentially drives metastasis and disease severity, depending on the state of local tissue homeostasis [12, 13].

This study aims to systematically evaluate all published necroptosis-related gene (NRG)-based prognostic signatures in cervical cancer and to integrate these findings with exploratory transcriptomic analyses to identify biologically relevant pathways and potential therapeutic targets, which have seen a widespread gain in recent interest [14, 15]. In addition, we examine the biological context of necroptosis in cancer to assess how this pathway may be leveraged to enhance anti-tumor immunity, particularly in combination with immunotherapeutic strategies, harnessing the increased antigenicity and adjuvanticity resulting from necroptosis [16]. Finally, published prognostic scores designed upon NRGs are systematically investigated as a way of identifying points of potential therapeutic intervention, providing a basis for generating novel biological hypotheses.

### The roles of necroptosis in cancers

Necroptosis has been implicated in both tumor suppression and tumor progression, highlighting its context-dependent role in cancer biology [3, 17, 18]. For example, in cervical [19–21] and prostate [22] cancers, higher levels of necroptotic effector proteins have been associated with improved outcomes. Similarly, necroptosis has been identified as a protective factor in colorectal cancer [23] and is not only beneficial, but a requirement for the success of immunotherapy in triple-negative breast cancer [24]. These observations highlight that several cancer therapies rely on the activation of immunogenic cell death for therapeutic success and durable treatment responses [16].

Conversely, in [25], esophageal [26], and pancreatic cancers [27], increased necroptosis and the resulting inflammatory signaling are associated with metastasis, poor treatment response, and worse prognosis [28]. Aberrant necroptosis proteins interrupt immune surveillance and lead to cancer progression [29]. This dual role of necroptosis is further discussed in later parts of this article in the context of necroptosis-related prognostic genes.

In cervical cancer, current evidence suggests that necroptosis is more frequently associated with better prognosis [19–21], reflecting its role in enhancing immune infiltration in poorly immunogenic tumors. This positions necroptosis as a promising candidate mechanism for overcoming immune evasion, one of the recognized hallmarks of cancer [30], that has formed the basis for some of the most successful and prolific cancer therapies marketed to date, including immune- and cell therapies. Necroptosis thus aligns well with the hallmark of immune evasion in its potential to reverse this paradigm, but also into the dysregulated cell death pathways and the newer description of dysregulated cellular metabolism: distinct, but interlinked hallmarks [30].

Mechanistically, RIPK3 is a central regulator of whether a cell can enter necroptosis [4, 31, 32]. RIPK3 expression is often silenced in cancer cells through epigenetic mechanisms such as a promotor methylation or post-transcriptional modifications [33], which can prevent necroptotic cell death. Restoration of RIPK3 expression using hypomethylating agents, has been proposed as a potential therapeutic avenue to re-sensitize tumor cells to immunogenic cell death [33]. However, the effects of such interventions appear to be context dependent, with both tumor-suppressive and therapy-resistance-associated outcomes reported. For example, the re-introduction of RIPK3 in a prostate cancer model reduced tumor cell proliferation *in vivo* [22], while in non-small cell lung cancer (NSCLC), RIPK3 restoration was linked to chemoresistance [34]. Other instances of the protective role of RIPK3 upregulation have been reported in colon cancer [18], lymphoma [35], and acute myeloid leukemia [36], where increased necroptosis, subsequently enhanced tumor control. Taken together, these findings highlight necroptosis as a complex and context-dependent process in cancer, with potential therapeutic relevance that depends on careful modulation of its activity within the tumor microenvironment [3, 37]. This is also true for other necroptotic machinery, including MLKL, which has recently been shown to be central in cancers’ adaptation towards immunotherapy resistance [38]. This was also on the epigenetic level as rescue was achievable through silencing and in other studies, for example through exogenous delivery of MLKL machinery [39].

The above are well described, canonical intervention points for the pathway of necroptosis, but further research is required into the interplay of these and other cellular processes which may have overlapping functionalities or synergies. Such pathways are hypothesized and discussed later in this article.

### Necroptosis and the tumor microenvironment in cervical cancer

Cervical cancer remains a major global health burden and is the fourth most common malignancy in women worldwide, occurring in increasingly younger patients, especially those of working and child-bearing age, highlighting the importance of less toxic and more durable management strategies [2, 40]. Despite advances in multimodality treatments including surgery, radiotherapy, cisplatin-based chemotherapy, and more recently, immunotherapy, clinical outcomes remain suboptimal for a substantial proportion of patients. In particular, limited immune cell infiltration has been consistently associated with poorer prognosis and reduced responsiveness to immune checkpoint inhibitors [41].

These observations have led to increased interest in characterization of “cold” and “ hot” tumor immune microenvironments in cervical cancer, as well as in strategies aimed at enhancing tumor immunogenicity [42, 43]. Approaches that promote immune cell recruitment and activation are considered key to improving the efficacy of immunotherapy via improvements in the antigenicity and the adjuvanticity of cervical tumors, increasing their chances of being naturally and durably cleared by the immune system with fewer adverse effects [37]. In this context, necroptosis represents a potential mechanism to enhance anti-tumor immunity.

As a regulated form of inflammatory cell death, necroptosis promotes the release of DAMPsand tumor antigens, thereby facilitating immune recognition. Cervical tumors are frequently characterized by relatively low immune infiltration, suggesting that induction of immunogenic PCD pathways may be beneficial in converting these tumors into more immune-responsive phenotypes [44]. However, this is not uniform across patients. Molecular heterogeneity exists between cervical cancer patients, revealing subgroups with simultaneously increased necroptosis-associated signaling and higher immune infiltration. This variability suggests that the role of necroptosis in cervical cancer is context-dependent and alludes to its influence on both prognosis and therapeutic response [9].

Given these observations, necroptosis-related gene (NRG) signatures have emerged as potential tools for patient stratification. Systematically evaluating these signatures could therefore unearth biological insights informing both prognostic mechanisms and potential therapeutic targets.

## Systematic review and (meta-)analysis of necroptosis-related gene signatures in cervical cancer

Advances in high-throughput transcriptomic profiling have enabled the development of molecular prognostic models based on NRGs across multiple cancer types, including cervical cancer [45]. These models, commonly constructed using RNA sequencing data and feature selection approaches, such as least absolute shrinkage and selection operator (LASSO) regression, aim to stratify patients according to observed survival outcomes. These signatures are then tested as predictors and validated as such.

Numerous NRG-based prognostic signatures have been reported in a range of cancers of varying primary location and characteristics. These signatures, also called risk scores, are designed using clinical transcriptomics data from publicly available datasets in most cases are validated in a group’s own dataset. Cervical cancer has received a fair amount of attention in this area [46–52], but the biological interpretation and consistency thereof across studies remain limited. In particular, overlap between identified genes and convergence on shared biological pathways have not been systematically assessed, restricting their translational application.

To address this, we performed a systematic review of published NRG-based prognostic models in cervical cancer and conducted a transcriptomic cross-study consensus analysis of the reported gene signatures. The recurrent genes and biological pathways derived from published signatures have formed the basis for investigation of new transcriptomic insights and will be instrumental in the design of new hypotheses informing compelling future experimentation and investigation.

### Methodology

This study was conducted in accordance with the Preferred Reporting Items for Systematic Reviews and Meta-Analyzes (PRISMA) guidelines to enable thorough, transparent, and unbiased reporting [53]. The review protocol was pre-registered in the PROSPERO database (ID CRD420251136495).

#### Search strategy and selection process

**Fig. 2:**
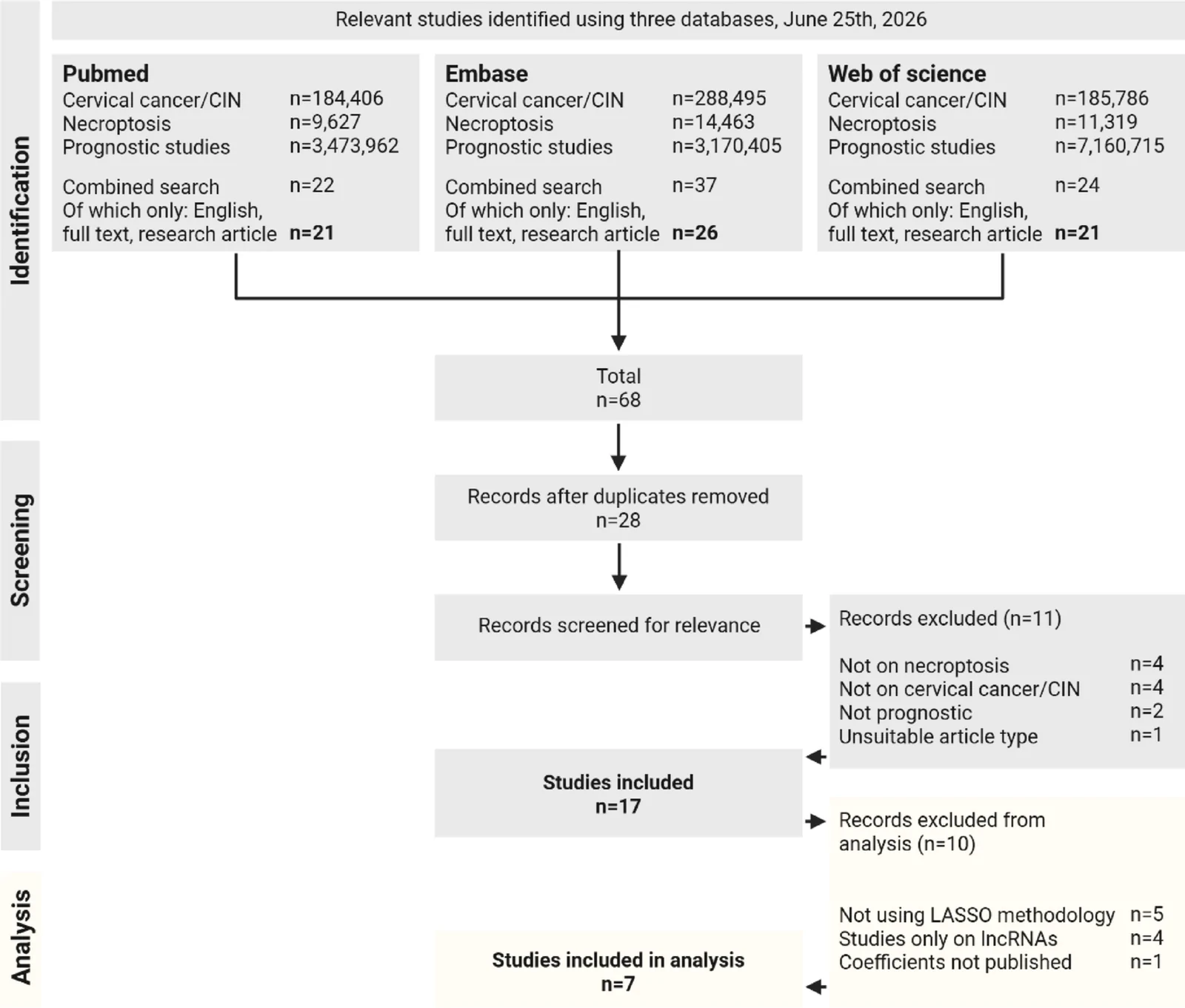
PRISMA flow chart. Database queries are shown, as well as search results, inclusion and exclusion criteria, and finally, included papers both for review and analysis. PRISMA: Preferred Reporting Items for Systematic Reviews and Meta-Analyzes.

A systematic literature search was performed in Pubmed (NCBI), Embase (Elsevier), and Web of Science (Clarivate) to identify studies reporting NRG-based prognostic signatures in cervical cancer. Exact search queries with text strings can be viewed in Supplementary Table S1A–C, as updated on June 25th, 2026. Study selection was conducted according to predefined inclusion and exclusion criteria, and the overall selection process is summarized in the PRISMA flow chart below (Fig. 2). Quality/bias appraisal of the studies included for analysis was done using the Newcastle-Ottawa scale tool for quality evaluation of non-randomized studies (Fig. S1A–B) [54].

Studies were eligible if they (i) investigated cervical cancer, (ii) reported prognostic signatures based on NRGs, and (iii) utilized transcriptomic data. NRGs were defined based on established pathway components, including RIPK3 and MLKL, as well NRG sets derived from pathway databases such as the Kyoto Encyclopedia of Genes and Genomes (KEGG) database (hsa04217) [45, 47, 51, 55], and Gene Ontology (GO) analyses [52, 56] related to necroptosis processes [57].

To enhance comparability across studies, we focused our analysis on prognostic models constructed using RNA sequencing data in combination with least absolute shrinkage and selection operator (LASSO) methodology, a widely used approach for feature selection in high-dimensional transcriptomic datasets. In the included studies,candidate genes were identified through differential expression analyses comparing a cancer dataset against a non-tumor dataset (e.g. TCGA and GTEx or own datasets), followed by model construction using LASSO regression.

#### Data harmonization and cross-study consensus analysis

Genes identified and their LASSO risk score coefficients were extracted from each study. This was followed by a transcriptomic cross-study consensus (meta-)analysis to identify overlapping genes across studies and to assess their relative contributions to reported risk scores. To facilitate comparison, raw LASSO coefficients were symmetrically scaled (anchored at 0) across studies to maintain the weight and direction (sign) of their relative contributions to the risk score as follows: relative to the local minimum and maximum of the LASSO hazard score per study, coefficients were assigned a relative value with same sign such that the maximum is 1, minimum is −1, and the midpoint is 0, by dividing each coefficient by the within-study maximum absolute coefficient magnitude. For each individual feature (*j*) within each individual study (*k*), let *β*_*j, k*_ represent the LASSO regression coefficient and β^*^_j, k_ the scaled coefficient, where *m* represents any feature in the set when searching for the maximum in study *k*:

$$\:{{\beta\:}^{*}}_{j,k}=\frac{{\beta\:}_{j,k}}{{\mathrm{m}\mathrm{a}\mathrm{x}}_{m}\left|{\beta\:}_{m,k}\right|}$$

Raw and scaled coefficients are reported in Supplementary Table S2, along with the MS Excel code used.

Genes were then grouped based on their frequency of occurrence. Gene sets were constructed based on frequency of occurrence (e.g. genes present in ≥ 2 studies; listed in Supplementary Table S3) and their potential prognostic relevance was assessed in a comparative manner. To compare the prognostic relevance of the new gene sets, the raw RNA expression value for each gene was multiplied by the mean of the symmetrically scaled coefficients for each respective gene, then summed to calculate a risk score. These risk scores were assigned to their respective patients and Kaplan-Meier curves were generated with the patient population being split at the median (into high and low risk score groups) for maximal reproducibility. Alongside this, the continuous concordance index and time-dependent AUC have been calculated as true measures of predictive power. These were calculated in R (v4.4.3) using the “survival” and “timeROC” packages.

Functional relationships among the identified genes were further explored using the STRING and KEGG databases, which provided insights into the biology underlying our findings.

### Results

A total of 68 relevant articles were identified across Pubmed, Embase, and Web of Science, of which 28 were duplicates. After title and abstract screening, 17 full-text English articles met the predefined inclusion criteria. Following additional selection for using RNA sequencing and LASSO-based prognostic modeling, seven studies were retained for comparative analysis (Fig. 2).

All included studies were published between 2015 and 2024 and focused on the development of necroptosis-related prognostic signatures in cervical cancer. The results of this literature search are reported in Table 1. Most studies derived candidate genes from publicly available transcriptome datasets, primarily The Cancer Genome Atlas (TCGA). Study characteristics and included gene signatures are summarized in Table 1. Of studies utilizing unique cohorts and reporting clinical outcomes (entries 13–17), four out of five suggest that necroptosis reduction is a feature of more advanced disease.

**Table 1:** Search results and extracted data relating to the construction of an NRG-based prognostic signature in cervical cancers.

#	Included in consensus analysis	Year	Refs.	Sample type	Databases used, patient selection (no selection unless stated)	Generation of prognostic signature	Genes, RNAs or proteins included	NRG source	Differential expression calculated against…	Findings based on prognostic signature (low vs. high risk)	Validation (if any)	Other
1	Y	2024	Xu et al. [57]	RNA, single-cell RNA	TCGA (*n* = 309: 01 A and 11 A. *n* = 283 01 A samples selected)	LASSO, KM, ROC	CD74, PGK1, NDRG1, LDHA, TPT1, TRIM29, ANXA2, ATP5B, S100A2	Genecards.org, KEGG, GO	Relevance score > 1 for necroptosis	Figure 2 – OS *p* < 0.0001	GSE44001 dataset, HPA histology database, cell culture and qPCR	Immune infiltration and ICB analysis, drug sensitivity analysis, nomogram, cellchat, GSEA, pseudotime, docking, HPA histology
2	Y	2023	Yang et al. [55]	RNA	TCGA (*n* = 273)	LASSO, KM, ROC, risk plot	SLC22A5, CHMP4C, VDAC1	Literature, KEGG hsa04217	GEO database, GSE44001 (*n* = 300)	Figure 4 – OS *p* = 1.352e−03	GSE44001, HPA, GEPIA	Nomogram, calibration curve
3	Y	2022	Zhan et al. [52]	RNA (incl lncRNA, miRNA)	TCGA (*n* = 303)	LASSO	ATRX, AXL, DDX58, IDH1, ITPK1, MAP3K7, SLC39A7, TARDBP, TNF, TNFRSF1A, TNFRSF1B, TNFSF10, TRIM11	KEGG, GO	GTEx	Figure 4 – OS *p* = 0.00194	qPCR	Immune infiltration and drug sensitivity
4	Y	2022	Sun et al. [51]	RNA	TCGA (*n* = 306)	LASSO	CAMK2A, CYBB, IL1A, IL1B, SLC25A5, and TICAM2	KEGG	TCGA & GTEx (TCGA *n* = 3, GTEx *n* = 19)	Figure 6 – OS *p* = 3.62e−05	GEO database GSE151666 & 206,224, IHC staining, GO analysis, Immune infiltration algorithm (TIMER & TISIDB)	TMB, MSI
5	Y	2022	Xing et al. [56]	RNA	TCGA (*n* = 306)	LASSO	TMP3, CHMP4C, EEF1A1, FASN, TNF, S100A10, IL1A, H1.2, SLC25A5, GLTP, IFNG, H2AC13, TUBB4B, AKNA, TYK2, H1.5	KEGG/GO (GO:0070266, 0097528, 0097527, 0060546, 0060545, 0060544, 1901026, 0062099, 0062100, 0097385, 1902445, 0062098)	Adjacent control samples (*n* = 3)	Figure 5 – OS *p* = 3.87e−05 (Validation set *p* = 5.75e−08)	International Cancer Genome Consortium (ICGC), The Cancer Imaging Archive (TCIA) database (*n* = 259)	TME score ESTIMATE, TMB, checkpoints, nomogram
6	Y	2022	Ma et al. [45]	RNA	TCGA, XENA-UCSC (*n* = 291)	LASSO	TNF, SLC25A5,VDAC1, FTH, IL1B, CHMP4C, BCL2, HIST1H2AE, HIST1H2AI	KEGG	GTEX or XENA-UCSC	Figure 5 – OS *p* < 0.001 [validation set n.s.]	Cross-validation and GEO (GSE44001) (*n* = 300)	Immune infiltration, CIBERSORT, EPIC, MCP counter, ssGSEA and ESTIMATE & Mutation & Drug sensitivity
7	Y	2024	Du et al. [47]	RNA	TCGA (incl: survival info and histologically confirmed CC: *n* = 303)	LASSO	IL1B, H2AZ1, H2AC14, TNFAIP3, CHMP4C, PLA2G4B, BCL2, CYBB, SLC25A5	KEGG	GTEX	Figure 4 OS *p* = 2.904 × 10e−5 (high risk worse off)	qPCR, GEO datasets (GSE30760, GSE52903 and GSE44001)	Enrichment (GO/KEGG/GSEA), nomogram, drug sensitivity prediction, CIBERSORT, TMB
8	N	2022	Zou et al. [58]	RNA	TCGA (*n* = 291)	LASSO	CXCL8, CLEC9A, Table 2	Literature, GSEA	UCSC Xena	Figure 7 – OS *p* = 1.08e−04	TME ESTIMATE algorithm, CIBERSORT, GSEA & KEGG, GO, GSE44001 (*n* = 300)	Drug sensitivity analysis
9	N	2022	Zhang et al. [59]	lncRNA	UCSC-XENA (*n* = 266, selected those with > 30 days of follow-up)	LASSO	AC009095.1, AC005332.4	KEGG, GENCODE	(n.a.)	Figure 2 – OS *p* = 0.00027	Cell lines (SiHa & HeLa, HUCEC & PANC-1), qPCR	Immune cells (xCell), GSEA (CAMOIP)
10	N	2022	Du X et al. [46]	lncRNA	TCGA (HTSeq-Counts & HTSeq-FPKM) (*n* = 265)	LASSO	DDN-AS1, DLEU1, RGS5, RUSC1-AS1, TMPO-AS1	Literature: Zhao et al. (2021) [60]	–	OS *p* < 0.001	Same set (bootstrap)	GSEA (immune) & independent factors, nomogram, TIMER, CIBERSORT, XCELL, QUANTISEQ, Drug sensitivity (oncoPredict)
11	N	2022	Lin et al. [61]	lncRNA, mRNA	TCGA (*n* = 304)	LASSO	MIR100HG, LINC00996, SNHG30, LINC02688, HCG15, TUBA3FP, MIAT, DBH-AS1, ERICH6-AS1, SCAT1, LINC01702	Literature, GSEA	Normal Cx tissues in TCGA	OS *p* < 0.001	Same set (bootstrap)	mRNA-lncRNA expression network constructed, nomogram, GSEA, KEGG/GO analysis, Immune, TMB
12	N	2023	Kong et al. [49]	lncRNA	TCGA (*n* = 304)	LASSO	AC092153.1, AC007686.3, LINC01281, AC009097.2, RUSC1-AS1	KEGG	Normal Cx tissues in TCGA and GTEx	OS *p* < 0.001 (Fig. 4)	Same set (bootstrap) and cervical epithelial cell line Ect1/E6E7, cervical cancer cell lines HeLa, SiHa, CaSki, and qPCR	Nomogram, PCA, functional enrichment, TMBB, Immuno and drug therapy response
13	N	2015	Ruan et al. [19]	Protein, IHC	Own cohort CC *n* = 54, FIGO I-II	Chi-sq and correlation	MLKL	–	Normal cervix *n* = 16	Higher MLKL survives longer (*p* = 0.036), negative correlation to grade and LN+	–	
14	N	2023	Guo et al. [62]	Retrospective, IHC	Own CC cohort (*n* = 100) FIGO I–III	Chi-sq and correlation	RIPK1	–	Normal cervix	PFS *p* = 0.001; OS *p* < 0.001 high expression of RIPK1 worse off, high RIPK1 expression correlates to FIGO II + III, LN+, and infiltration depth: *p* = 0.011, *p* = 0.001, *p* = 0.006 respectively (Table 2)	–	Also correlations found between RIPK1 and SCC antigen, Tumor size, and age (all +)
15	N	2023	Vogelsang et al. [21]	IHC	Own CC cohort (*n* = 250) FIGO I–IV	HR and correlation	RIPK1, RIPK3, pMLKL	–	–	All necroptotic proteins were expressed significantly less in higher grade tumors. RIPK1: OS *p* = 0.029, RFS *p* = 0.013. RIPK3: OS *p* = 0.01, RFS *p* = 0.004. RIPK1 + RIPK3+pMLKL: OS *p* = 0.019.	Cervical cancer cell lines HeLa, SiHa, CaSki	BrdU, C2 ceramide stimulation decreases survival but only if necroptosis is inhibited.
16	N	2024	Vogelsang [63]	IHC	Own CC cohort (*n* = 250) FIGO I–IV	HR and correlation	Heat shock protein 90	–	–	High HSP90 expression bodes well for survival and recurrence in cervical squamous cell carcinoma patients through involvement in PCD pathways.	–	Nuclear and cytoplasmic HSP90 signals tested for effect on patient survival (Kaplan-Meier).
17	N	2018	Li et al. [20]	RNA, protein	Own cohort CC *n* = 89, HPV+, FIGO I–IV	–	MLKL, (RIPK3)	—	Healthy controls *n* = 15	Higher FIGO = > lower MLKL, high MLKL = > better OS (*p* = 0.009) & DFS (*p* = 0.0003)	Cell lines (SiHa, HeLa, C33A, HaCaT, U937, Macrophages)	PBMC extraction, qPCR, WB, cytokines measured.

NRG, necroptosis-related genes; TCGA, The Cancer Genome Atlas; GTEx, The Genotype-Tissue Expression project; KEGG, Kyoto Encyclopedia of Genes and Genomes; GO, gene ontology; HPA, human protein atlas; GEO, gene expression omnibus; TMB, tumor mutational burden; MSI, microsatellite instability; TME, tumor microenvironment; GSEA, gene set enrichment analysis; LASSO, Least Absolute Shrinkage and Selection Operator; Kaplan-Meier; ROC, receiver-operating curve

#### Frequently reported genes and each one’s contribution to cervical cancer risk scores

To identify genes consistently associated with necroptosis-related prognostic signatures in cervical cancer, reported LASSO coefficients were collected, symmetrically scaled while preserving coefficient directionality, and averaged across studies. Validation using Kaplan-Meier analyses demonstrated that this scaling approach did not alter prognostic stratification, indicating that the scaling and normalization preserved the identical biological behavior of the original signatures and did not alter the data. Of the 309 patients included in the validation dataset, 194 contained survival data and could be used to investigate the prognostic scores in this paper.

**Fig. 3:**
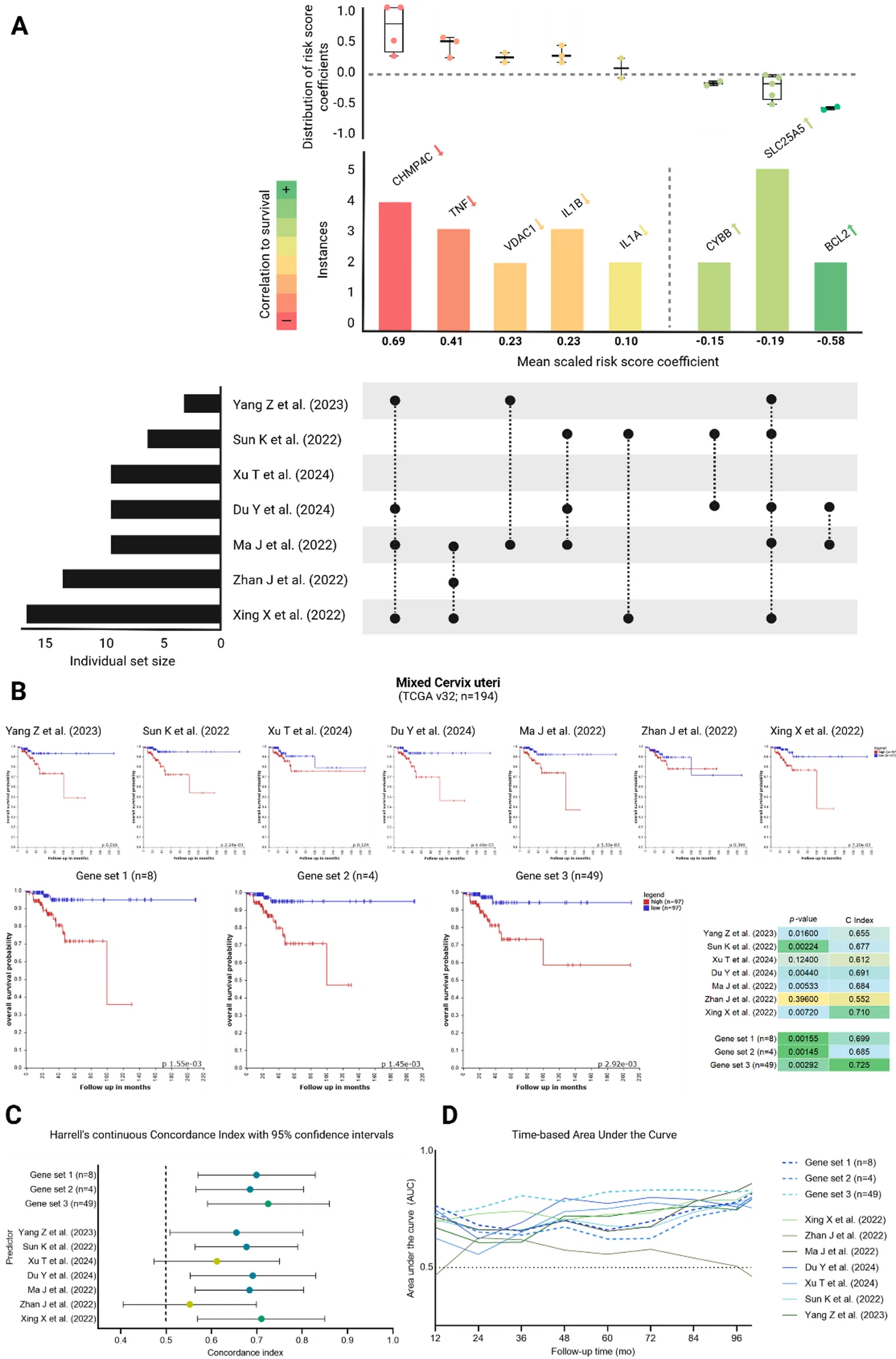
**A** UpSet plot showing overlap in differentially expressed necroptosis-related genes (DENRGs) with symmetrically scaled mean prognostic significance in cervical cancer. Genes overlapping in at least two studies are shown alongside their +/- correlation to overall survival, denoted as risk score coefficient. The distributions of the scaled coefficients from literature are shown above. **B** Kaplan-Meier (KM) graphs showing the survival probability of the TCGA v32 cervix uteri cohort, with group divisions based on the combined gene sets derived from literature; authors mentioned alongside KM graphs. The upper row of KMs represents the individual published groupings, while the lower row of KMs represents groupings based on the median risk score of gene set combinations. Gene sets: 1; all genes overlapping in at least two articles (*n* = 8), 2; all genes overlapping in more than two articles (*n* = 4), and 3; all genes reported across all included articles (*n* = 49). The p values are reported, showing that the smallest combined gene set gives the highest confidence in separating high and low risk groups in this cohort over a period of 200 months, alongside the Harrell’s continuous concordance index, showing the prediction accuracy of each risk score. **C** Forest plot showing the Harrell’s continuous concordance indices for each prediction model with 95% confidence intervals. **D** Time-based Area under the curve (AUC) graph for all prediction models, truncated at 100 months to avoid model instabilty.

Seven genes were recurrently identified across independent studies, including CHMP4C, TNF, VDCAC1, IL1B, IL1A, SLC25A5, CYBB and BCL2 (In full: Charged Multivesicular Body Protein 4C, Tumor Necrosis Factor, Voltage-dependent anion channel 1, Interleukin 1 Beta & Alpha, Solute Carrier Family 25 Member 5, Cytochrome B-245 Beta Chain, and B-cell Lymphoma 2, respectively; Fig. 3A) and summarized using an upset plot [64]. These genes were primarily associated with mitochondrial regulation, reactive oxygen species (ROS) generation, inflammatory signaling, membrane integrity, and immune modulation. Only genes identified in at least two independent studies were included in the overlap analysis. The distribution of scaled coefficients is shown in the upper area of Fig. 3A.

#### Generating a new consensus-derived prognostic score from the included studies

To investigate whether frequently occurring genes retained prognostic value when combined across studies, three consensus gene sets were generated based on the degree of overlap between published signatures: (1) genes present in at least two studies, (2) genes present in more than two studies, and (3) all genes identified across included studies. The consensus-derived gene sets were tested based on their combined risk scores on the TCGA Mixed Cervix Uteri (v32) cohort (Fig. 3B). These analyses were performed using the R2: Genomics Analysis and Visualization Platform (http://r2.amc.nl). Interestingly, the most restricted consensus gene set (Gene set 2) containing the smallest number of overlapping genes demonstrated the highest confidence in separating the risk-score groups by median (Fig. 3C; *p* = 0.00145), whereas the strongest prognostic performance by way of concordance index was seen in Gene set 3 (Fig. 3D; C-index = 0.725). Including the 49 genes with their coefficients performed better prognostically than all individual published signatures also tested in the same TCGA set, suggesting that recurrently identified genes may capture biologically robust necroptosis-associated prognostic information in cervical cancer.

#### Unraveling the biological roles of these genes using the STRING database

**Fig. 4:**
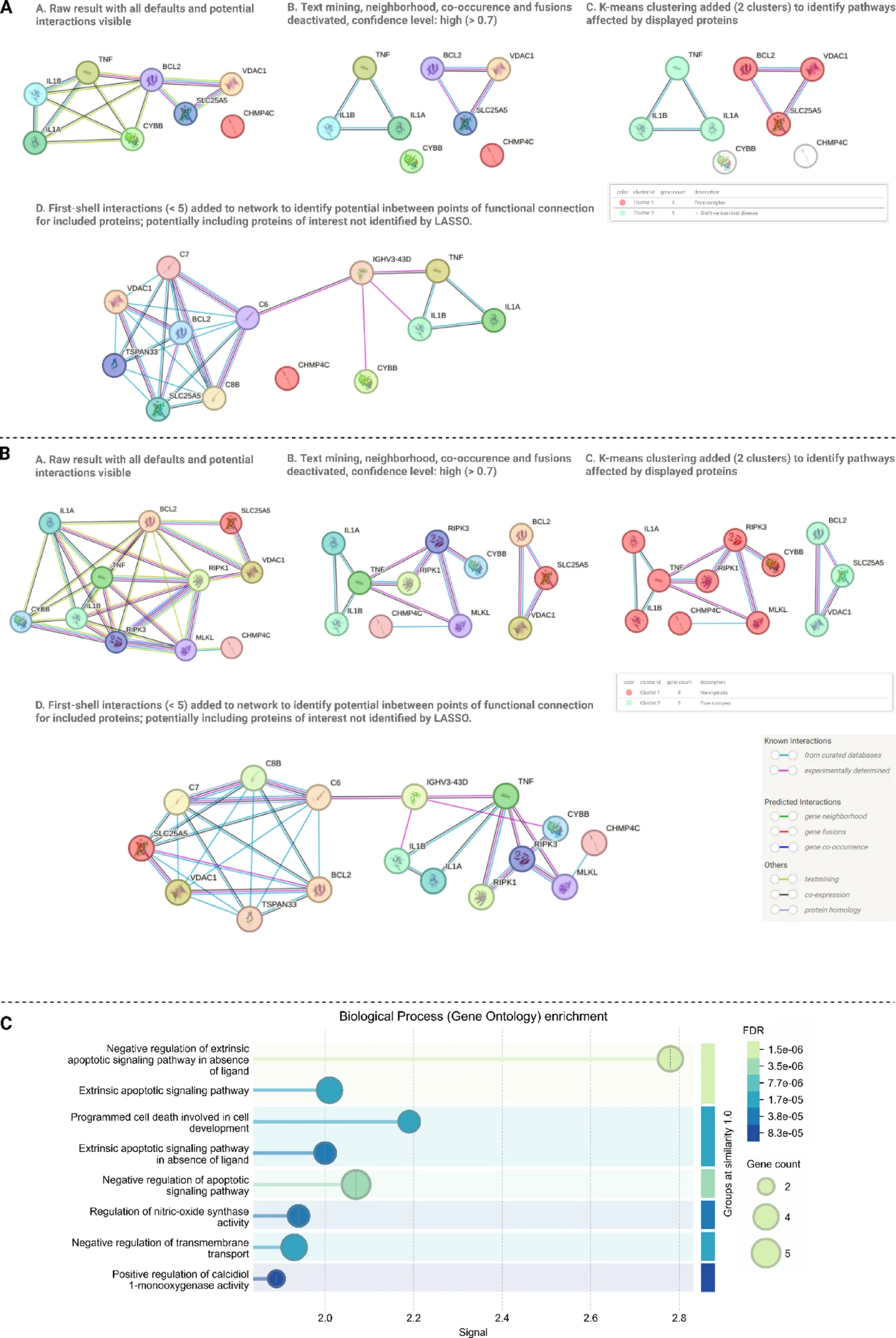
**A** STRING analysis for proteins identified by LASSO. **B** STRING analysis as before, with the classic necroptosis effector proteins added. **C** Gene ontology analysis from the STRING database showing most involved processes with the named genes, excluding the classic necroptosis machinery.

To further investigate the biological relationships between recurrently identified genes, protein-protein interaction analysis was performed using the STRING database version 12.0 [65]. A high confidence level of 0.7 was used while excluding text-mining, neighborhood, co-occurrence and fusion interactions. Interaction analysis demonstrated functional clustering around necroptotic signaling, mitochondrial metabolism, ROS regulation, inflammatory signaling, and membrane pore formation (Fig. 4A, B). K-means clustering and first-shell interaction analysis identified additional interacting proteins, including complement-associated components such as C6, C7 and C8B.

Gene ontology enrichment analysis demonstrated interactions of the identified proteins with known biological processes, such as extrinsic apoptosis signaling, mitophagy, inflammatory responses, lipid metabolism, and angiogenesis-related pathways (Fig. 4C). Enrichment analyses was performed using STRING gene ontology annotations at the highest similarity setting (≥ 1), with terms ranked according to signal strength. Collectively, these findings suggested that necroptosis-related prognostic signatures in cervical cancer are associated with broader biological networks involving mitochondrial regulation, oxidative stress, inflammatory signaling, and immune modulation, extending beyond canonical necroptotic signaling pathways.

### Discussion

In this systematic review and transcriptomic cross-study consensus analysis, we have identified necroptosis-related genes associated with prognosis in cervical cancer from consensus in published literature and explored their underlying mechanistic roles within the tumor microenvironment. Across the included studies, consensus genes converged on pathways involving mitochondrial regulation, ROS generation, inflammatory signaling, and interactions, both inhibitory and stimulatory, between PCD pathways. Importantly, many genes associated with favorable prognosis appeared to promote conditions permissive for necroptosis or suppress competing non-immunogenic PCD pathways, especially apoptosis. As such, the prognostic significance of necroptosis-related signaling appears highly context dependent. While increased necroptotic activity generally correlated with improved survival outcomes in cervical cancer, opposite associations have been reported in other tumor types [44]. These discrepancies likely reflect differences in tumor immune composition, stromal architecture, basal inflammatory signaling, and the balance between beneficial transient anti-tumor immunity and chronic tumor-promoting inflammation. Necroptosis should therefore not be viewed as universally favorable or unfavorable; rather as a context-dependent regulator of tumor-immune interactions.

**Fig. 5:**
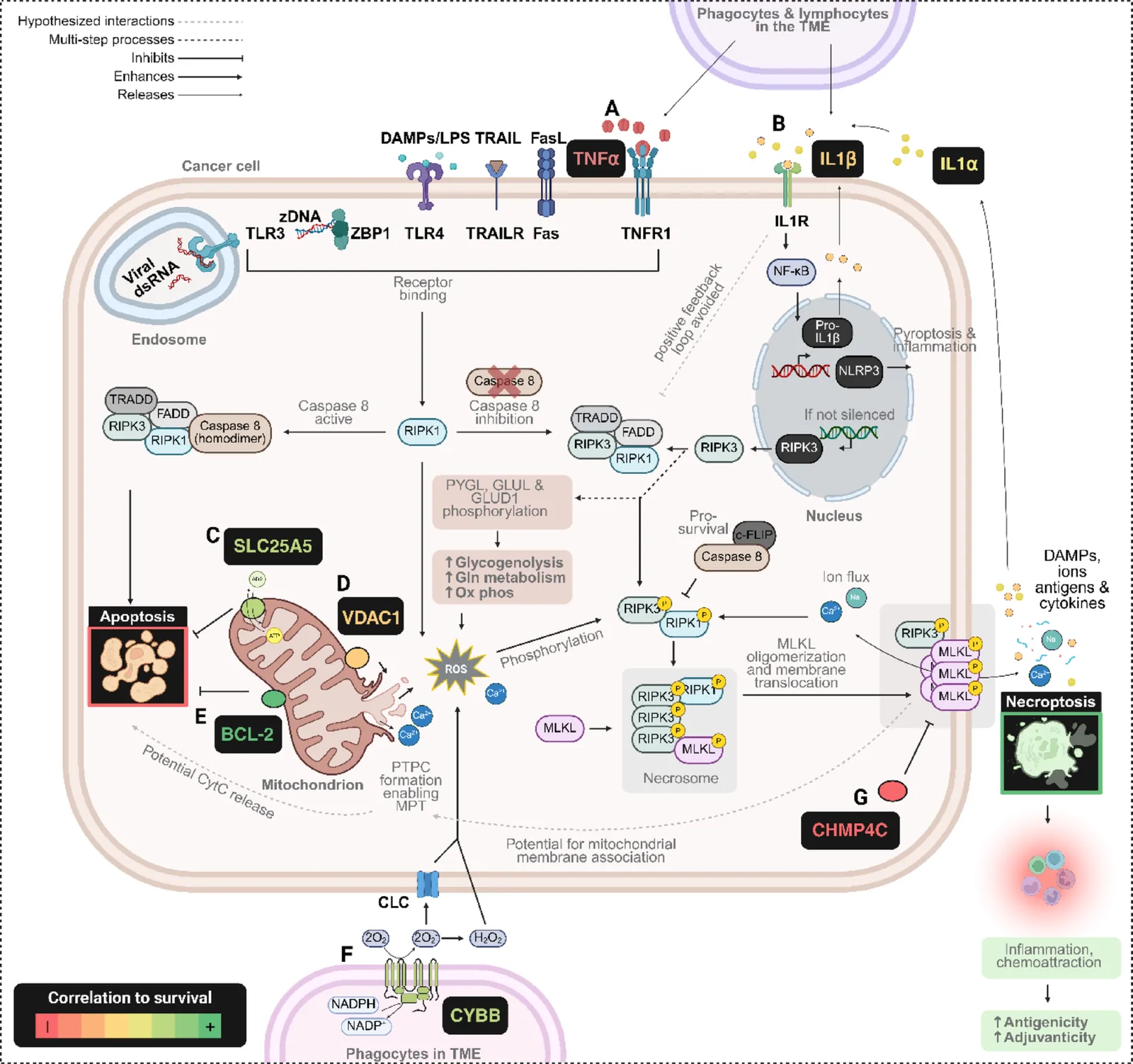
The signaling overview of the eight identified tumor microenvironment proteins from consensus in literature with the biggest effect on survival in a necroptosis related context. Their effects on and interactions with necroptotic signaling are hypothesized here. The classical necroptosis cascade is initiated by the binding of RIPK1 to RIPK3, the phosphorylation and oligomerization of RIPK3 to form the necrosome, the attraction of MLKL, and the translocation of this complex to the plasma membrane, where cell contents, including DAMPs and antigenic material leak out of the cell, causing necrosis-like cell death, inflammation, and immune attraction. This is necessarily enabled by the inactivation of caspase 8 and the presence of ROS. **A **TNFα was identified to have a negative effect on survival in cervical cancer. This is thought to its activation of apoptosis through receptor interactions, and due to caspase 8 activity blocking necroptosis. In the case where caspase 8 is inhibited, necroptosis can proceed upon receptor binding of TNFα. **B** IL1α & β, proinflammatory cytokines, also had a negative effect on survival. Upon binding to the IL1 receptor, inflammation is induced through the NLRP3 inflammasome axis and pyroptosis. This also includes the release of IL1β, which inhibits the interaction between RIPK1 and RIPK3, preventing necroptosis. This is thought to be a regulatory mechanism by which constitutive activation is avoided, preventing chronic inflammation. Furthermore, IL1α is one of the inflammatory DAMPs released upon MLKL-pore-mediated permeabilization. **C** SLC25A5 assists in the transport of ADP in, and ATP out, of the mitochondria. Its activity inhibits apoptosis and it has been linked to better survival in cervical cancer patients. **D** VDAC1 regulates the PTPC formation, allowing leakage of Ca^2+^ ions and ROS, essential for necroptosis to occur. **E** BCL2, the well known apoptosis inhibitor, was also associated with better survival in cervical cancer patients. **F** CYBB, bound to the membrane of phagocytes in the TME, induces ROS as a killing mechanism, which can enhance necroptosis. CYBB is also known as NOX2 and correlated to better survival in cervical cancer patients. **G** CHMP4C has a role in repairing membrane pores, thus counteracts necroptosis. It is associated with shortened survival. From the above the hypothesis is that any protein enhancing necroptosis-related processes aligned with improved survival probability, while proteins enabling apoptosis were associated with shorter survival. Multivesicular Body Protein 4C; CYBB: Cytochrome B-245 Beta Chain; CytC: Cytochrome C; DAMPs: Danger-AssociatedMolecular Patterns; dsRNA: Double-stranded Ribonucleic Acid; FADD: Fas-Associated protein with Death Domain; Fas: FirstApoptosis Signal Receptor; FasL: Fas Ligand; GLUD1: glutamate dehydrogenase 1; GLUL: glutamate-ammonia ligase; IL1B:Interleukin 1 Beta; IL1R: Interleukin 1 Receptor; LPS: lipopolysaccharide; MLKL: Mixed Lineage Kinase Domain-Like protein;MOMP: Mitochondrial Outer Membrane Permeabilization; MPT: Mitochondrial Permeability Transition; NADP: NicotinamideAdenine Dinucleotide Phosphate (Oxidized); NADPH: Nicotinamide Adenine Dinucleotide Phosphate (Reduced); NLRP: NODLikeReceptor Family Pyrin domain containing; PTPC: Permeability Transition Pore Complex; PGYL: glycogen phosphorylase;RIP: Receptor-Interacting Protein; ROS: Reactive Oxygen Species; SLC25A5: Solute Carrier Family 25 Member 5; TLR3: Toll-Like Receptor 3; TLR4: Toll-Like Receptor 4; TNF: Tumor Necrosis Factor; TNFR: Tumor Necrosis Factor Receptor; TRADD:TNF Receptor Type 1-Associated Death Domain protein; TRAIL: TNF-Related Apoptosis-Inducing Ligand; TRAILR: TNF-RelatedApoptosis-Inducing Ligand Receptor; VDAC1: Voltage-Dependent Anion-Selective Channel 1; ZBP1: Z-DNA Binding Protein 1;zDNA: Z-DNA (Left-handed DNA)

We have designed a hypothetical cell-signaling interaction diagram showing where the consensus proteins identified in this review may interact with canonical necroptotic signaling. It is important to bear in mind that these proteins were identified for their prognostic significance and not their direct effects on necroptosis. Collectively, these findings support a model in which proteins which promote mitochondrial ROS generation, suppress apoptosis, or facilitate inflammatory immune activation are generally associated with improved survival in cervical cancers, whereas proteins limiting plasma membrane permeabilization or redirecting signaling toward alternative death pathways are associated with poorer outcomes (Fig. 5).

### Mitochondrial regulation, ROS signaling, and the balance between apoptosis and necroptosis

A recurring theme emerging from the identified genes is the central role of mitochondrial metabolism and ROS generation in regulating necroptotic susceptibility. Relationships between the two have been previously explored in depth [6, 29, 66, 67], and we have investigated further cancer type-specific underlying mechanistic insights.

Several consensus genes, including CHMP4C, SLC25A5, VDAC1, CYBB, and BCL2, either directly regulate mitochondrial function or influence oxidative stress responses. Although mitochondrial involvement is not considered an absolute requirement for necroptosis, mitochondrial ROS production can promote shifts from apoptosis to necroptosis under conditions of insufficient oxidative metabolism, partly through assisting the phosphorylation of RIPK3 [67].

RIPK3 kinase activity extends beyond MLKL phosphorylation and may regulate metabolic pathways through interactions with proteins including PYGL (glycogen phosphorylase), GLUL (glutamate-ammonia ligase), and GLUD1 (Glutamate Dehydrogenase 1), increasing glycogenolysis and glutaminolysis, promoting ROS formation [4, 68, 69]. These fundings suggest that necroptosis is closely linked to metabolic adaptation rather than an isolated cell death mechanism. This may be particularly relevant in cervical cancer, where tumors commonly exist within hypoxic and metabolically stressed microenvironments characterized by insufficient metabolism, hypoxia, acidification and aerobic glycolysis (the Warburg effect) [70, 71]. Under these conditions, mitochondrial dysfunction and ROS generation may support both necroptotic signaling and immunogenicity through enhanced DAMP release.

MLKL-mediated membrane disruption further amplifies this relationship. MLKL oligomerization has also been associated with mitochondrial membrane permeabilization, leakage, loss of membrane potential, and potential downstream inflammasome activation following ROS release [67, 72]. However, mitochondria may simultaneously function as downstream regulators, limiting constitutive necroptotic signaling through interactions with RIPK3 [67, 73]. These regulatory mechanisms are biologically important, as constitutive necroptotic activation may become detrimental, leading to uncontrolled inflammatory signaling. The rescue of caspase-8 knockout lethality through RIPK3 deletion demonstrated the consequences of uncontrolled necroptosis [74]. For this reason, therapeutic strategies aiming to exploit necroptosis will crucially require transient rather than constitutive activation to maximize anti-tumor immunity while minimizing chronic inflammation. Taken together with the classical necroptotic effector proteins mentioned earlier in this paper, the conditions created by the proteins identified below should remain in a coherent balance to prevent overexposure to inflammatory cell death, or to transiently induce it as part of the anti-cancer immunity cycle [67, 75].

### Proteins negatively associated with survival

#### CHMP4C

CHMP4C belongs to the Endosomal Sorting Complex Required for Transport III (ESCRT-III) complex family involved in membrane remodeling, budding, cytokinesis, exosome release and diverse intra- and extracellular membrane-based trafficking [76]. In necroptosis, ESCRT proteins counteract MLKL-mediated membrane damage through membrane repair processes following pore formation [77], and may even form MLKL-loaded vesicles, blocking necroptosis induction [44]. This activity may reduce the beneficial consequences of necroptosis in poorly infiltrated tumors by limiting DAMP release and reducing immune activation. Consistent with our hypothesis, higher CHMP4C expression was repeatedly associated with poorer outcomes in cervical cancer [45, 47, 55]. Similar associations have also been reported in melanoma [45] pancreatic [77], prostate [78], and lung cancers [79].

#### TNF

TNFα is one of the principal initiators of inflammatory signaling and necroptotic activation [80]. However, TNFα signaling alone is insufficient to induce necroptosis, requiring simultaneous RIPK1 activation and caspase-8-inhibition [6]. Without these conditions, TNFα signaling preferentially induces immunologically silent apoptosis or activates persistent cell-survival pathways [45, 52, 56]. These findings suggest that cytokine abundance alone may not predict beneficial necroptotic activity; instead, pathway context appears critical. TNF activity seems to be a central mechanism of action of Second mitochondria-derived activator of caspase (Smac)-mimetic therapies, as endogenous TNF expression is induced, enhancing local immune responses and responses to chemotherapy [81].

Interestingly, a link has also been shown between TNFAIP3 expression and poorer prognosis [47], and while this is in agreement with the above TNF-related findings, this is not captured in Fig. 3A. TNFAIP proteins are known positive mediators of tumor progression in many cancer types [82], especially types 1, 3 and 8 [83, 84]. Further, TNFR expression was identified as a positive contributor to patient survival as part of a necroptosis-related risk score designed for esophageal carcinoma, conflicting with scores designed on cervical cancer patients, highlighting different needs for different cancer types based on their local immune situation [50].

#### VDAC1

VDAC1 regulates mitochondrial permeability transition pore opening and influences mitochondrial calcium transport and ROS generation [85]. Increased VDAC1 expression was associated with poorer prognosis in cervical cancer cohorts [45, 55], despite mechanistic evidence linking VDAC1 to necroptosis [86–88]. (Fig. 3A) This apparent contradiction may reflect VDAC1’s pleiotropic role. While mitochondrial permeabilization can promote ROS-dependent necroptosis, it may simultaneously support apoptosis, metabolic adaptation, autophagy and tumor progression [31, 89]. Therefore, VDAC1 likely functions as a context-dependent regulator rather than a direct necroptosis factor.

One study investigating lncRNAs inhibiting necroptosis in liver cancer cells under hypoxic conditions identified VDAC1 as the regulator of this inhibition through increased mPTP opening. Inhibition was removed upon knockdown, but further investigation is needed on this mechanism, as mPTP opening may also contribute to increased autophagy and mitophagy [90]. It was also found that induction of VDAC1 through oxygen-glucose deprivation and reoxygenation correlated to an induction of both necroptosis and apoptosis as identified by measurement of activated (p)MLKL and cleaved caspase-3 in neuronal cells. This was partly rescued by an inhibitor of VDAC1 (VBIT-12), showing the impact of VDAC1 expression on (necroptotic) cell death induction [91]. Another study has suggested that VDAC1 associates with MLKL to assist with translocation to the mitochondrial membrane, leading to pore formation and leakage of Ca and ROS, and necroptosis [92].

VDAC1 was also identified as part of an NRG-based prognostic gene signature in breast [48, 93], melanoma [45], and lung cancer settings [94], being associated with a higher risk and poorer patient prognosis. *In vitro* VDAC1 knockdown caused a significant reduction in growth and migration of breast cancer cells [93]. While seemingly contradictory, in the multicellular *in vivo *situation, immunogenic PCD pathway induction may be more telling than total cell death observed in a tumor cell monoculture for the TME-wide immunogenic picture. This points to the fact that the regulation and downstream effects of VDAC1 expression and necroptosis cannot be accurately studied within classical tumor cell line models. Immune and/or stromal compartments are essential to properly recapitulate the effects of these pathways on survival of cells* in vitro*.

#### IL1α & IL1β

IL1α and IL1β are pleiotropic pro-inflammatory cytokines. Where IL1α is more commonly associated with inflammation and is considered an alarmin or DAMP [95], IL1β is a context-dependent tumor-immunity downregulator [96], implicated in tumor progression through angiogenesis and epithelial-to-mesenchymal transition, while potentially driving immunosuppression in the TME through desmoplasia formation and immunosuppressive TAM polarization [97, 98].

Across multiple cancers, including cervical, increased IL1β expression is associated with poor prognosis and/or metastasis [51, 99–101]. Mechanistically, IL1β occupies a dual role within necroptosis. Although necroptosis promotes active IL1β release [102], IL1β-signaling may simultaneously suppress RIPK1-RIPK3 interactions, limiting further (constitutive) necroptotic activation [73]. This likely avoids a -wide positive feedback mechanism preventing uncontrolled inflammatory amplification, as chronic activation may promote tumor progression. Interestingly, IL1α was not uniformly reported to be beneficial or not to the survival of cervical cancer patients [51, 56], pointing to the influence of the inflammatory state of a patient’s local TME [95], and leaving this correlation up to context.

### Proteins positively associated with survival

#### CYBB

CYBB, also known as nicotinamide adenine dinucleotide phosphate (hydrogen) (NADPH) oxidase 2 (NOX2), is involved in ROS production through membrane-localized NADPH oxidase activity and plays a central role in antimicrobial immune responses [47]. CYBB has been observed in a range of tissues and is known for its involvement in oxygen catalysis generating ROS [103]. In cervical cancer, higher CYBB expression is associated with improved survival and increased immune infiltration signatures, including CD8+ T-cell enrichment [2, 51]. CYBB is also a favorable biomarker in cervical cancer: expression of CYBB mediates IFN-based defenses against HPV in cervical cancer and indicates higher survival probability [104].

ROS generated through CYBB may contribute to necroptosis by facilitating RIPK1 autophosphorylation and necrosome assembly [105, 106]. Although ROS dependency remains context-dependent, these findings support the broader observation that oxidative stress pathways may support beneficial immunogenic cell death. The link has been widely described [6, 74, 107–109].

An interesting example from an *in vivo* rat model was that the inhibition of RIPK1 by necrostatin-1 significantly reduced the expression of NOX2 and showed protective effects against doxorubicin-induced cardiotoxicity [110]. Doxorubicin is an anthracycline in use for its ability to induce immunogenic cell death [111]. Necroptosis is also a form of immunogenic cell death, drawing a potential link between necroptosis, RIPK1 inhibition and NOX2 expression.

#### SLC25A5

SLC25A5 encodes the protein ANT2, a mitochondrial ADP/ATP antiporter across the mitochondrial membrane (ADP in, ATP to cytoplasm). Apoptosis induction has been associated with suppression of ANT2 activity, thus increased SLC25A5 expression may indirectly favor non-apoptotic death programs, including necroptosis [51]. This may explain why SLC25A5 repeatedly appeared as a favorable prognostic marker in cervical cancer cohorts [45, 47, 51, 56]. In acute myeloid leukemia, also known to feature immunosuppression within the TME, SLC25A5 has a positive association with prognosis [112]. Interestingly, opposite associations were reported in esophageal carcinoma [50], supporting the context-dependent nature of inflammatory cell death pathways. This could be due to esophageal cancers typically having infiltrated (“hot”) immune microenvironments [113], where cervical cancers, generally speaking, do not [114].

#### BCL2

BCL2 suppresses apoptosis through inhibition of pro-apoptotic proteins, including Bax and Bak [115]. Despite its classical anti-apoptotic role, increased BCL2 expression was consistently associated with improved survival in cervical cancer [45, 47]. It is plausible that suppression of apoptosis may shift stressed cells toward alternative death pathways, including necroptosis [3]. However, the suppression of apoptosis may also be a key mechanism by which cells resist chemotherapy-induced cytotoxicity; thus, the shift toward induction of necroptosis could enhance inflammatory signaling and immune cell recruitment, particularly in immunologically cold tumors, and reduce chemotherapy resistance [115].

### STRING database analysis: insights from k-means clustering, first-shell inclusions, and gene ontology enrichment

STRING (Search Tool for the Retrieval of Interacting Genes/Proteins) interaction analysis was performed to contextualize the identified consensus genes within broader biological networks and identify potential functional relationships beyond canonical necroptotic signaling. Although these analyses are influenced by database coverage and published literature bias, they provide useful hypotheses regarding pathway interactions and biological convergence.

The interaction networks revealed clustering around necroptotic signaling, membrane pore formation, mitochondrial regulation, and inflammatory pathways, supporting the notion that the identified prognostic genes converge on interconnected biological processes rather than acting independently. Expansion of the interaction network with first-shell interactions revealed complement proteins interacting with necroptotic signaling, including C6, C7 and C8B, suggesting potential links between membrane pore formation, calcium signaling and necroptotic regulation that require further investigation.

Gene ontology enrichment analysis demonstrated enrichment of processes associated with regulation of extrinsic apoptotic signaling, inflammatory signaling, innate immunity, and membrane transport. The analysis was generated at the highest similarity level (≥ 1), with terms ranked by signal. Notably, negative regulation of extrinsic apoptotic signaling was most strongly represented, reinforcing the dependence of necroptosis on apoptosis inhibition. The innate immunity and inflammatory aspects further highlight the context-dependent nature of necroptosis and also the deep involvement thereof with immunity, where inflammatory signaling may contribute to both anti-tumor immunity and tumor-promoting effects.

Overall, these interaction and enrichment analyses support the biological coherence of the identified prognostic genes and reinforce the central role of mitochondrial regulation, inflammatory signaling, and cell death pathway crosstalk in cervical cancer.

### Therapeutic implications

The collective findings suggest that therapeutic induction of necroptosis may represent a strategy to improve anti-tumor immunity in cervical cancer, particularly in tumors with poor immune infiltration. However, sustained necroptotic signaling may promote chronic inflammation, angiogenesis, stromal remodeling, and metastatic progression. Therefore, therapeutic approaches should prioritize transient induction of immunogenic cell death rather than prolonged pathway activation.

The widely agreed-upon context dependency [44], observed across tumor types, further suggests that patients stratification based on immune composition and tumor microenvironment characteristics may be necessary before implementing necroptosis-directed interventions.

Given the potential for necroptosis induction to synergize with immunotherapies, recent studies have investigated this combination in therapeutic settings [44, 116]. Relatively few instances exist where necroptosis induction has been deployed alongside immunotherapies, especially checkpoint inhibition. This is likely due to the slow clinical adoption of specific “necroptosis inducers” and the potential for off-target effects when these agents are delivered in an untargeted manner. However, Wu et al., through screening and knockdown studies, have identified that inhibition of the protein Mouse Double Minute 2 (MDM2) increases necroptosis by preventing ubiquitination and proteasomal degradation of the necrosome protein complex. MDM2 knockdown significantly reduced lymphoma growth in mice and synergized with anti-PD-1 treatment to significantly reduce tumor burden in a melanoma model [117]. In another study, Zhang et al. investigated a novel EGFR inhibitor molecule which was found to induce necroptosis in diverse human *in vitro* cancer models, while also generating a potent adaptive immune response and showing significant synergy with PD-1 blockade in a mouse model of melanoma [118]. Clinical data further support a potential role for necroptosis in response to checkpoint inhibition. Sax et al. have drawn a link between necroptosis and checkpoint inhibitor resistance by investigating the survival probability of malignant melanoma patients undergoing treatment with anti-PD-1/anti-CTLA-4. Patients with higher MLKL expression in tumor tissue, indicative of greater necroptosis competence, showed significantly better survival than those with low expression of necroptosis machinery [38]. Together, these findings support further investigation of necroptosis as a potential component of effective immunotherapy strategies and highlight the potential for therapeutic combinations with immune checkpoint inhibitors.

Several other therapeutic options directly targeting or synergizing with necroptosis, have been explored [37, 119, 120]. Readers are referred to these publications for more a in-depth study of therapeutic implications of necroptosis.

### Strengths and limitations

This study combined systematic literature review with cross-study transcriptomic analysis, allowing identification of consensus prognostic markers across independently developed signatures. Integration with network analyses and pathway interpretation further improved biological contextualization.

Several limitations should be acknowledged. First, included studied relied heavily on retrospective transcriptomic datasets, primarily derived from TCGA. Although studies used overlapping TGCA cervical cancer cohorts, differential expression analyses were performed on a diverse range of normal tissue cohorts, including GTEx datasets, GEO cohorts, independent patient cohorts from healthy patients, or adjacent healthy tissue. Nevertheless, a similar data source may have introduced some bias into the analysis. Second, prognostic signatures varied in methodology, gene selection, and validation approaches, and independent validation in external cohorts will be required to establish their generalizability. Such validation is planned as a future step. Thus, mechanistic conclusions remain hypothetical until further testing can confirm or deny the statements made in this paper. Experimental validation falls beyond the scope of this systematic review, but remains highly interesting for the future of cancer immunotherapy. We recommend wet-lab validation of the findings of this paper for further progress. Lastly, we have utilized the median as the cutoff for risk score groups in the Kaplan-Meier curves to ensure consistency across studies and with the source publications, which all used the median. Although alternative data-driven cutoff methods could result in different groupings, applying study-specific thresholds would introduce substantial heterogeneity and potential bias in cross-study comparisons. We therefore considered the median cutoff the most appropriate approach for this analysis. We acknowledge that the median may not represent the most biologically informative cutoff; however, it provides a consistent and unbiased basis for comparison.

## Conclusion and outlooks

The findings of this study suggest that necroptosis-related prognostic signatures in cervical cancer converge on common biological themes involving mitochondrial regulation, ROS generation, inflammatory signaling, and interactions between PCD pathways, including reliance on apoptosis inhibition. It is evident that the usefulness of necroptosis as part of a therapeutic strategy is highly context-dependent, where its induction is generally desirable as an immune induction strategy in immunologically “cold” tumors, but undesirable in “hot” or inflamed tumors. In the latter situation, necroptosis induction indicates poor clinical outcomes. Further, necroptosis upregulation should be transient and not chronic, as constitutive activation leads to inflammation-associated complications.

In the included cervical cancer cohorts, genes promoting conditions permissive for necroptosis are generally associated with improved survival, whereas genes limiting membrane disruption or favoring alternative pathways such as apoptosis or autophagy tended to be associated with poorer outcomes. While highly interesting, these claims remain hypothetical, pending functional experimental validation.

These findings support further investigation of necroptosis as both a prognostic framework and a therapeutic target in cervical cancer, particularly within strategies aiming to enhance anti-tumor immunity.

## Supplementary Information

Below is the link to the electronic supplementary material.

Supplementary Material 1

## Data Availability

All data handled in this manuscript was obtained from publicly available sources.

## Abbreviations

ADP: Adenosine diphosphate
ATP: Adenosine triphosphate
BCL2: B-cell lymphoma 2
CHMP4C: Charged multivesicular body protein 4C
CYBB: Cytochrome B-245 beta chain
CytC: Cytochrome C
DAMPs: Danger-associated molecular patterns
dsRNA: Double-stranded ribonucleic acid
FADD: Fas-associated protein with death domain
Fas: First apoptosis signal receptor
FasL: Fas ligand
GLUD1: Glutamate dehydrogenase 1
GLUL: Glutamate-ammonia ligase
IL1B: Interleukin 1 beta
IL1R: Interleukin 1 receptor
LPS: Lipopolysaccharide
MLKL: Mixed lineage kinase domain-like protein
MOMP: Mitochondrial outer membrane permeabilization
MPT: Mitochondrial permeability transition
NADP: Nicotinamide adenine dinucleotide phosphate (oxidized)
NADPH: Nicotinamide adenine dinucleotide phosphate (reduced)
NLRP: NOD-like receptor family pyrin domain containing
PTPC: Permeability transition pore complex
PGYL: Glycogen phosphorylase
RIP: Receptor-interacting protein
ROS: Reactive oxygen species
SLC25A5: Solute carrier family 25 member 5
TLR3: Toll-like receptor 3
TLR4: Toll-like receptor 4
TNF: Tumor necrosis factor
TNFR: Tumor necrosis factor receptor
TRADD: TNF receptor type 1-associated death domain protein
TRAIL: TNF-related apoptosis-inducing ligand
TRAILR: TNF-related apoptosis-inducing ligand receptor
VDAC1: Voltage-dependent anion-selective channel 1
ZBP1: Z-DNA binding protein 1
zDNA: Z-DNA (left-handed DNA)
