# Supplementary material for: Necroptosis as a key player in cervical cancer progression and management: a systematic review and transcriptomic cross-study consensus analysis
Source: Apoptosis. 2026 Oct 11;31(11):244. doi: 10.1007/s10495-026-02440-w (PMC13654877; doi:10.1007/s10495-026-02440-w)
Supplement: Supplementary file 1 [file 10495_2026_2440_MOESM1_ESM.docx]

**Supplementary material**

***Necroptosis as a key player in cervical cancer progression and management ‒ a systematic review*** ***and transcriptomic cross-study consensus analysis***

**Table S1:** Search queries and terms entered into databases, with search results as of June 25^th^ 2026.

**A**

| PUBMED \| 2026-06-25 | | |
| --- | --- | --- |
| # | Search query | Results |
| #1 – CC OR CIN | ((("Uterine cervical neoplasms"[Mesh] OR "cervix lesion"[tiab] OR "cervical lesion*"[tiab] OR “cervical carcinoma”[tiab] OR “cervical squamous cell carcinoma”[tiab] OR “cervical adenocarcinoma”[tiab] OR (("Cervix uteri"[Mesh] OR "cervi*"[tiab]) AND ("Neoplasms"[Mesh] OR "cancer"[tiab] OR "neoplas*"[tiab] OR "carcinoma"[tiab] OR "malignan*"[tiab] OR "tumor*"[tiab] OR "tumour*"[tiab]))) OR ("Uterine cervical dysplasia"[Mesh] OR “Cervical Intraepithelial Neoplasia”[tiab])) | 184,406 |
| #2 – Necroptosis | ("Necropt*"[tiab]) | 9,627 |
| #3 – Prognostic studies | ("Predict*"[tiab] OR "prognos*"[tiab] OR "biomarker"[tiab]) | 3,473,962 |
| #4 – Combined | #1 AND #2 AND #3 | 22 |
| #5 – Inclusion | (“English”[Language] AND “Full text”[Filter])  #4 AND #5 | 22 |
| #6 – Exclusion | ("review"[Publication Type] OR "meta-analysis"[Publication Type] OR “systematic review”[Publication Type])  #4 AND #5 NOT #6 | 21 |
| Finalized search string | | |
| ((("Uterine cervical neoplasms"[Mesh] OR "cervix lesion"[tiab] OR "cervical lesion*"[tiab] OR "cervical carcinoma"[tiab] OR "cervical squamous cell carcinoma"[tiab] OR "cervical adenocarcinoma"[tiab] OR (("Cervix uteri"[Mesh] OR "cervi*"[tiab]) AND ("Neoplasms"[Mesh] OR "cancer"[tiab] OR "neoplas*"[tiab] OR "carcinoma"[tiab] OR "malignan*"[tiab] OR "tumor*"[tiab] OR "tumour*"[tiab]))) OR ("Uterine cervical dysplasia"[Mesh] OR "Cervical Intraepithelial Neoplasia"[tiab]))) AND ((("Predict*"[tiab] OR "prognos*"[tiab] OR "biomarker"[tiab])) AND (("Necropt*"[tiab]))) AND (("English"[Language] AND "Full text"[Filter])) NOT ("review"[Publication Type] OR "meta-analysis"[Publication Type] OR "systematic review"[Publication Type]) | | |

**B**

| EMBASE \| 2026-06-25 | | |
| --- | --- | --- |
|  | Search query | Results |
| #1 – CC OR CIN | ‘Uterine cervical neoplasms’/exp OR ‘cervix lesion’:ab,ti,kw OR ‘cervical lesion*’:ab,ti,kw OR ‘cervical carcinoma’:ab,ti,kw OR ‘cervical squamous cell carcinoma’:ab,ti,kw OR ‘cervical adenocarcinoma’:ab,ti,kw OR ((‘Cervix uteri’/exp OR ‘cervi*’:ab,ti,kw) AND (‘Neoplasms’/exp OR ‘cancer’:ab,ti,kw OR ‘neoplas*’:ab,ti,kw OR ‘carcinoma’:ab,ti,kw OR ‘malignan*’:ab,ti,kw OR ‘tumor*’:ab,ti,kw OR ‘tumour*’:ab,ti,kw)) OR (‘Uterine cervical dysplasia’/exp OR ‘Cervical Intraepithelial Neoplasia’:ab,ti,kw) | 288,495 |
| #2 – Necroptosis | ('Necroptosis'/exp OR 'necroptosis':ab,ti,kw) | 14,463 |
| #3 – Prognostic studies | ('prediction'/exp OR 'prognosis'/exp OR 'biological marker'/exp OR 'prediction':ab,ti,kw OR 'prognosis':ab,ti,kw OR 'biological marker':ab,ti,kw) | 3,170,405 |
| #4 – Combined | #1 AND #2 AND #3 | 37 |
| #5 – Inclusion | (‘English’:la AND ‘Article’:it)  #5 AND #6 | 27 |
| #6 – Exclusion | (‘review’:it OR ‘meta-analysis’:it OR ‘systematic review’:it)  #4 AND #5 NOT #6 | 26 |
| Finalized search string | | |
| ('uterine cervical neoplasms'/exp OR 'cervix lesion':ab,ti,kw OR 'cervical lesion*':ab,ti,kw OR 'cervical carcinoma':ab,ti,kw OR 'cervical squamous cell carcinoma':ab,ti,kw OR 'cervical adenocarcinoma':ab,ti,kw OR (('cervix uteri'/exp OR 'cervi*':ab,ti,kw) AND ('neoplasms'/exp OR 'cancer':ab,ti,kw OR 'neoplas*':ab,ti,kw OR 'carcinoma':ab,ti,kw OR 'malignan*':ab,ti,kw OR 'tumor*':ab,ti,kw OR 'tumour*':ab,ti,kw)) OR 'uterine cervical dysplasia'/exp OR 'cervical intraepithelial neoplasia':ab,ti,kw) AND ('necroptosis'/exp OR 'necroptosis':ab,ti,kw) AND ('prediction'/exp OR 'prognosis'/exp OR 'biological marker'/exp OR 'prediction':ab,ti,kw OR 'prognosis':ab,ti,kw OR 'biological marker':ab,ti,kw) AND 'english':la AND 'article':it NOT ('review':it OR 'meta-analysis':it OR 'systematic review':it) | | |

**C**

| WEB OF SCIENCE \| 2026-06-25 | | |
| --- | --- | --- |
|  | Search query | Results |
| #1 – CC OR CIN | TS=(“Uterine cervical neoplasms” OR “cervix lesion” OR “cervical lesion*” OR “cervical carcinoma” OR “cervical squamous cell carcinoma” OR “cervical adenocarcinoma” OR ((“Cervix uteri” OR “cervi*”) AND (“Neoplasms” OR “cancer” OR “neoplas*” OR “carcinoma” OR “malignan*” OR “tumor*” OR “tumour*”)) OR (“Uterine cervical dysplasia” OR “Cervical Intraepithelial Neoplasia”)) | 185,786 |
| #2 – Necroptosis | TS=(”Necropt*”) | 11,319 |
| #3 – Prognostic studies | TS=("Predict*" OR "prognos*" OR "biomarker*") | 7,160,715 |
| #4 – Combined | #1 AND #2 AND #3 | 24 |
| #6 – Inclusion & Exclusion | LA=(English) AND DT=(Article)  #5 AND #6 | 21 |
| Finalized search string | | |
| ((((TS=((“Uterine cervical neoplasms” OR “cervix lesion” OR “cervical lesion*” OR “cervical carcinoma” OR “cervical squamous cell carcinoma” OR “cervical adenocarcinoma” OR ((“Cervix uteri” OR “cervi*”) AND (“Neoplasms” OR “cancer” OR “neoplas*” OR “carcinoma” OR “malignan*” OR “tumor*” OR “tumour*”)) OR (“Uterine cervical dysplasia” OR “Cervical Intraepithelial Neoplasia”)))) AND TS=(("Necropt*"))) AND TS=(("Predict*" OR "prognos*" OR "biomarker*"))) AND LA=(English)) AND DT=(Article) | | |

**Figure S1:** The Newcastle-Ottawa scale tool used to evaluate the quality of non-randomized studies (Wells, Shea et al. 2015)

**A:** Quality evaluation criteria

|  |  |
| --- | --- |

**B:** Outcomes of The Newcastle-Ottawa quality assessment for all analyzed studies. “n/a” was assigned if this criterion could not be applied to the current study.

| Ref | Selection | | | | | | | Comparability | | | Outcome | | |  | Score /6 | |
| --- | --- | --- | --- | --- | --- | --- | --- | --- | --- | --- | --- | --- | --- | --- | --- | --- |
|  | **1)** | | **2)** | **3)** | | **4)** | | **1)** | | | **1)** | | **2)** | **3)** |  |  |
| Yang Z et al. 2023 | * | * | | | n/a | | n/a | |  | * | | * | * | * | ****** | 6 |
| Sun K et al. 2022 | * | * | | | n/a | | n/a | |  | * | | * | * | * | ****** | 6 |
| Xu T et al 2022 | * | * | | | n/a | | n/a | |  | * | | * | * | * | ****** | 6 |
| Du Y et al 2022 | * | * | | | n/a | | n/a | |  | * | | * | * | * | ****** | 6 |
| Ma J et al. 2022 | * | * | | | n/a | | n/a | |  | * | | * | * | * | ****** | 6 |
| Zhan J et al. 2022 | * | * | | | n/a | | n/a | |  | * | | * | * | * | ****** | 6 |
| Xing X et al. 2022 | * | * | | | n/a | | n/a | |  | * | | * | * | * | ****** | 6 |

**Table S2:** Unscaled and scaled coefficients as reported

|  | | **Yang Z et al.** | **Zhan J et al.** | | **Sun K et al.** | | **Xing X et al.** | | **Ma J et al.** | | **Du Y et al.** | | *Mean* |
| --- | --- | --- | --- | --- | --- | --- | --- | --- | --- | --- | --- | --- | --- |
| **CHMP4C** | |  |  | |  | |  | |  | |  | |  |
| Unscaled as reported | | 0.066 |  | |  | | 0.190 | | 0.635 | | 0.045 | |  |
| Scaled | | 1.000 |  | |  | | 0.496 | | 1.000 | | 0.262 | | *0.69* |
| **TNF** | |  |  | |  | |  | |  | |  | |  |
| Unscaled as reported | |  | 0.2339 | |  | | 0.204 | | 0.143 | |  | |  |
| Scaled | |  | 0.474 | |  | | 0.533 | | 0.225 | |  | | *0.41* |
| **VDAC1** | |  |  | |  | |  | |  | |  | |  |
| Unscaled as reported | | 0.0102 |  | |  | |  | | 0.189 | |  | |  |
| Scaled | | 0.155 |  | |  | |  | | 0.298 | |  | | *0.23* |
| **IL1B** | |  |  | |  | |  | |  | |  | |  |
| Unscaled as reported | |  |  | | 0.4098 | |  | | 0.234 | | 0.019 | |  |
| Scaled | |  |  | | 0.207 | |  | | 0.368 | | 0.110 | | *0.23* |
| **CYBB** | |  |  | |  | |  | |  | |  | |  |
| Unscaled as reported | |  |  | | -0.369 | |  | |  | | -0.021 | |  |
| Scaled | |  |  | | -0.186 | |  | |  | | -0.122 | | *-0.15* |
| **SLC25A5** | |  |  | |  | |  | |  | |  | |  |
| Unscaled as reported | | -0.003 |  | | -0.276 | | -0.120 | | -0.286 | | -0.001 | |  |
| Scaled | | -0.046 |  | | -0.139 | | -0.313 | | -0.451 | | -0.006 | | *-0.19* |
| **BCL2** | |  |  | |  | |  | |  | |  | |  |
| Unscaled as reported | |  |  | |  | |  | | -0.385 | | -0.096 | |  |
| Scaled | |  |  | |  | |  | | -0.607 | | -0.558 | | *-0.58* |
|  | |  | |  | |  | |  | |  | |  |  |
|  | **MS Excel formula:** | | | | | | | | | | | | |
|  | *= “coefficient” / MAX(ABS(MIN(“all coefficients within a study”)), ABS(MAX(“all coefficients within a study”)))* | | | | | | | | | | | | |

**Table S3:** Genesets compiled from systematic review and genomic analysis part 3

| Geneset 1 | Genes overlaping in ≤ 2 articles | BCL2  CHMP4C | IL1B  IL1A | TNF SLC25A5 | VDAC1 | CYBB |
| --- | --- | --- | --- | --- | --- | --- |
| Geneset 2 | Genes overlapping in >2 articles | CHMP4C | IL1B | SLC25A5 | TNF |  |
| Geneset 3 | all genes reported across all included articles | AKNA  ANXA2  ATP5F1B  ATRX  AXL  BCL2  CAMK2A  CD74  CHMP4C  CYBB | DDX58  EEF1A1  FASN  FTH1  GLTP  H1-2  H1-5  H2AC13  H2AC14  H2AC8 | H2AZ1  IDH1  IFNG  IL1A  IL1B  ITPK1  LDHA  MAP3K7  NDRG1  PGK1 | PLA2G4B  S100A10  S100A2  SLC25A5  SLC39A7  TARDBP  TICAM2  TNF  TNFAIP3  TNFRSF1A | TNFRSF1B  TNFSF10  TPM3  TPT1  TRIM11  TRIM29  TUBB4B  TYK2  VDAC1 |
